# High Doses of Halotolerant Gut-Indigenous* Lactobacillus plantarum* Reduce Cultivable Lactobacilli in Newborn Calves without Increasing Its Species Abundance

**DOI:** 10.1155/2017/2439025

**Published:** 2017-05-17

**Authors:** Alexander Rodriguez-Palacios, Henry R. Staempfli, J. Scott Weese

**Affiliations:** ^1^Division of Gastroenterology and Liver Disease, Case Western Reserve University School of Medicine, Cleveland, OH 44106, USA; ^2^Digestive Health Research Institute, Case Western Reserve University School of Medicine, Cleveland, OH 44106, USA; ^3^Department of Clinical Studies, Ontario Veterinary College, University of Guelph, Guelph, ON, Canada N1G 2W1; ^4^Department of Pathobiology, Ontario Veterinary College, University of Guelph, Guelph, ON, Canada N1G 2W1

## Abstract

To elucidate the ecological effect of high oral doses of halotolerant (resistant to table salt) indigenous-gut bacteria on other commensals early in life, we conducted a culture-based study to quantify the effect of intestinal* Lactobacillus plantarum* strain of bovine origin (with remarkable aerobic growth capabilities and inhibitory activity against* Escherichia coli* O157:H7 and F5) on clinical health and gut lactobacilli/coliforms in newborn calves. In a double-blind placebo-randomized trial twelve colostrum-fed calves, consecutively born at a farm, were fed* L. plantarum* within 12 hours from birth at low (10^7-8^ CFU/day) or high concentrations (10^10-11^) or placebo (q24 h, 5 d; 10 d follow-up). We developed a 2.5% NaCl-selective culture strategy to facilitate the enumeration of* L. plantarum*-strain-B80, and tested 384 samples (>1,152 cultures).* L. plantarum*-B80-like colonies were detected in a large proportion of calves (58%) even before their first 24 hours of life indicating endemic presence of the strain in the farm. In contrast to studies where human-derived* Lactobacillus* LGG or* rhamnosus* had notoriously high, but short-lived, colonization, we found that* L. plantarum* colonized stably with fecal shedding of 6 ± 1 log_10_·g^−1^ (irrespective of dose, *P* > 0.2). High doses significantly reduced other fecal lactic acid bacteria (e.g., lactobacilli, *P* < 0.01) and slightly reduced body weight gain in calves after treatment. For the first time, a halotolerant strain of* L. plantarum* with inhibitory activity against a human pathogen has the ability to inhibit other lactobacilli* in vivo* without changing its species abundance, causing transintestinal translocation, or inducing clinical disease. The future selection of probiotics based on halotolerance may expand therapeutic product applicability.

## 1. Introduction

Probiotics are widely available in the market and high doses are anecdotally recommended. However, for most probiotics, the actual dose-dependent effect on intestinal health and other gut bacteria remains largely uncertain, especially with regard to gut-indigenous strains. Although there is no consensus on what “high-dose” means for a microbial fed product, the World Health Organization and the Food and Agricultural Organization of the United Nations define probiotics as “live microorganisms which when administered in adequate amounts confer a ‘health benefit' on the host” [1]. Such definition emphasizes the relevance of the nonspecified high “amount” because beneficial outcomes in earlier studies were elicited only when “high doses” of probiotic bacteria were used [2].

Sequence-based methods for evaluating the gut 16s rDNA microbiome have shown great promise and have become increasingly common in digestive diseases research [3]. However, they do not recapitulate community composition of simple culture-based mock communities [4]. The great variability recognized in 16s rDNA microbiome studies is primarily introduced by variability relevant to the target gene and region sequences, sequencing platform, with less impact by the method of DNA extraction [4–7]. Further, 16s rDNA microbiome analysis cannot distinguish between dead and alive bacteria and has suboptimal sensitivity, that is, limit of high throughput sequencing detection of low abundant bacteria to identify and quantify microorganisms at the strain level [8].

To elucidate the ecological effect of high oral doses of specific gut-indigenous bacterial strains on other commensals early in life, here we conducted a preliminary culture-based study to quantify the effect of an aerobic (aerotolerant anaerobe with remarkably optimal growth at room air) gut-indigenous* Lactobacillus plantarum* strain (which we previously isolated from calves with the most potent inhibitory activity against* Escherichia coli* O157:H7 and F5 and ability to survive acidic conditions and high bile salt concentrations; see [9]) on the clinical health and gut lactobacilli and coliform populations in the feces and intestinal mucosa of newborn bottle-fed calves, given the clinical relevance of mucosa-associated microbiota [10].

To contribute to the understanding of the role of lactic acid bacteria (LAB, i.e.,* Lactobacillus*,* Bifidobacterium Enterococcus* and* Pediococcus *spp.) in animal health and food safety, we previously characterized >100 LAB isolates from cattle and found from a potentially therapeutic perspective two types of extreme profiles: one inhibitory of pathogenic* Escherichia coli* and one stimulatory [9]. Ten percent of the LAB isolates significantly inhibited* E. coli* strains responsible for serious diseases in calves and humans (types F5 and O157:H7), while another 10% of isolates promoted (>2-fold) their growth in vitro [9, 11]. Among the inhibitory strains, a calf-derived strain of* L. plantarum* (isolate B80, herein “*Lplant*-B80”) had optimal properties to be suitable for preclinical dose-dependent studies in animals. The strain had excellent aerobic growth and acid resistance and its colony was morphologically distinct and was frequently isolated from the small intestinal mucosa of calves [9]. Because* E. coli* O157:H7 is a serious foodborne human pathogen widely present in the cattle industry [11], it is important to identify mechanisms to prevent colonization and supershedding in animals, especially if therapeutic/preventive mechanisms could be administered with the diet.

Among natural inhabitants of plants,* L. plantarum* is the most promising organism for commercial therapeutic microbiology in humans because they have strong immune modulatory properties [12] and could be added to plant-based diets. To contribute to the understanding of dose-effect responses of gut-indigenous bacteria in animals, the objective of this blind placebo-controlled randomized trial was to determine the safety and quantitative effect of low- and high doses of gut-derived* L. plantarum Lplant*-B80 on other intestinal LAB and coliforms when given orally to newborn colostrum-fed healthy calves.

## 2. Materials and Methods

### 2.1. Animals and Husbandry

Healthy neonatal Holstein Friesian male calves (12–24 hours old, fed > 4 L of fresh colostrum within 6 h of birth) were included in this preclinical study under the approval of the Animal Care Committee of the University of Guelph. The study was conducted in winter and used animals that originated from the same experimental farm this* L. plantarum* strain was originally isolated from [9]. To prevent clustering and increase study heterogeneity, animals were consecutively enrolled as they were born (the first two born of the week; 1-2/week). Available animals were randomly assigned to three groups upon arrival to the research facility (preassigned ballots “in a hat” strategy), before the study commenced (placebo and low- and high-dose of* Lplant*-B80), which resulted in three random uneven cohorts (*n* = 3, 5, and 4). No further animals were available from the same farm for this preclinical trial. Calves were kept in individual pens. Hay and water (ad libitum), and the bedding (wood shavings), were daily replaced to minimize fecal-oral recycling of microorganisms.

### 2.2. Inoculum

*Lplant*-B80 was fed to the animals (body weight average per group: placebo, 44.0, high-dose, 44.8, and low-dose, 45.2 kg, *P* > 0.2) as freeze-dried pellets containing 10^7-8^ or 10^10-11^ CFU (low- and high-dose) per day, following a triple blinded approach at pellet assignment, follow-up, and statistical analysis. Placebo pellets were prepared with 0.5 g of freeze-dried lactose-free powder milk to resemble the* Lplant*-B80 pellets. All pellets were administered dissolved in fresh whole milk derived from in-house milking Holstein cows (*n* = 3) that had not received antibiotics for >60 days, once a day in the morning for 5 days. Animals were monitored for additional 10 days. For proper body weight gain analysis, the milk volume fed daily corresponded to 10% of the animal body weight in kilograms, divided into two feedings (q12h). BSL2-practices were implemented to prevent cross-contamination and infections with infectious pathogens of neonatal calves. Feeding bottles were washed, disinfected for 10 minutes with 10% aqueous sodium hypochlorite, and rinsed with sterile water within 30 minutes of use.

### 2.3. Preparation of* Lactobacillus plantarum* Pellets for Blinded Trial

To prepare* Lplant*-B80 freeze-dried pellets, 50 mL of de Man, Rogosa, and Sharp (MRS) broth (Oxoid) was inoculated with 24-hour colonies of* Lplant*-B80 and incubated at 37°C for 24 hours which was inoculated onto 1-L MRS broth. Bacteria were harvested after 24 h by centrifugation (4400 ×g, 15 minutes, 4°C) and washed 1x with phosphate buffered saline. For freeze-drying, 6% dextran and 0.9% of NaCl solution (Gentran 70®) was used to resuspend bacteria (1 : 1 w/v). The suspensions (aliquoted as 2.5 mL in 4-mL glass test tubes) were frozen at −80°C for 24 h prior to freeze-drying at −50°C. The resulting freeze-dried pellets were stored sealed in the tubes with a rubber cap at −80°C and used within 3 weeks of preparation. The concentration of live* Lplant*-B80 was verified in random pellets. Culture of leftover milk from feeding bottle confirmed viability at ingestion. A set of sealed tubes containing the pellets were left at room temperature (23°C; 70% humidity) and monitored for bacterial viability at 6, 12, and 48 months.

### 2.4. Follow-Up and Samples

Daily physical examinations were performed to all animals by a specialist in internal medicine. Body weight was determined prior to feeding on days 1 and 2 to monitor neonatal health and hydration status and on days 6 and 15 to assess weight gain. Rectal temperature, appetite, and attitude were assessed daily. Fresh fecal samples were collected by digital palpation, directly from the rectum before and after inoculation on days 1–3, 5–7, 9, 11, 13, and 15 (*n* = 10). Animals were euthanized on day 15 to determine persistent bacterial systemic translocation by collecting gastrointestinal mucosal specimens (rumen ventral-caudal sac, pylorus, ileum, cecal apex, small colon, and ileocecal lymph node) for enumeration of* L. plantarum,* total LAB, and fecal coliforms, as the importance of mucosal associated microbiota is increasingly relevant in our understanding of intestinal inflammation [13–15]. All samples were stored at −80°C and processed together at the end of the feeding trial.

### 2.5. Total Anaerobic Lactic Acid Bacteria and Aerobic Coliforms

Immediately after collection, feces were homogenized, weighted, and aliquoted (5–10 g) for storage and freeze-drying. To adjust for the variable water content of feces in neonatal animals, all bacterial enumeration analyses were adjusted to dry matter and normalized to CFU·gr^−1^ of dry feces. The water content was determined via freeze-drying of 2 fecal aliquots per sampling day. Enumeration of fecal LAB and coliforms was conducted with the spread-plate method and 10-fold serial dilutions. MRS agar was used for enumeration of LAB (anaerobic, 48 h, 37°C) and MacConkey agar for coliforms (aerobic, 24 h, 37°C).

### 2.6. Isolation Protocol for Recovery of* Lplant*-B80

We previously reported that* Lplant*-B80 grows in MRS broth adjusted with hydrochloric acid to pH 4 and that it was aerobically able to grow on NaCl-MRS agar [9]. Here we combined those two strong properties to optimize the isolation and differentiation of* Lplant*-B80-like colonies from other LAB in fecal/intestinal samples. Compared to a rifampicin-resistant subclone we prepared for this study (twenty 24 h-incubation passages with increasing antibiotic concentrations), testing indicated there was no need for selective antibiotics. Optimization experiments with HCl and NaCl were conducted with feces spiked with* Lplant*-B80. The limit of detection from experimentally inoculated feces was 10^2-3^ CFU/g. We also verified the consistent selective ability of this method during coculture with 20 other random LAB isolates that represent the spectrum of the LAB collection that was previously derived from the same farm and temporal frame [9].* Lplant*-B80 colonies were distinct from other LAB (i.e., yellowish, 4-5 mm round flat colonies, Gram-positive short rods) after aerobic incubation on 2.5% NaCl-MRS agar at 37°C for 3–5 days.

### 2.7. Quantitative and Qualitative Detection of* Lplant*-B80

For quantitation* (CFU estimation)* in feces, tenfold serial dilutions were prepared using pH4-MRS broth, aerobically incubated at 37°C for 2 h (optimal time without affecting* Lplant*-B80 CFUs in validation experiments), and spread plated (100 *μ*L) for aerobic incubation onto 2.5%-NaCl-MRS agar at 37°C for three days. For qualitative enrichment* (presence/absence)* of* Lplant*-B80, the inoculated pH4-MRS broths were incubated for additional 24 h; then, 10 *μ*L of the broth was streaked onto 2.5%-NaCl-MRS agar.

For tissues, the detection of* Lplant*-B80 in mucosal surfaces was based on qualitative triplicate analysis* (presence/absence)* using broth. In brief, after removing the intestinal content by gently pressure-flushing the surface with PBS via a 50-mL syringe/18 G needle, 1 cm^2^ × 0.5 mm mucosa was aseptically dissected for aerobic enrichment in 5 mL of pH4-MRS broth as described for feces. Lymph nodes were cultured after cutting longitudinally 1-2 mm slices, 0.5 cm apart. Every sample batch had three autoclaved intestinal specimens concurrently tested as negative controls.* Lplant*-B80-like colonies were enumerated after 5 days of aerobic incubation, purified by subculture on 2.5%-NaCl-MRS agar, tested biochemically, and a subset confirmed with Sanger sequencing of 16S rRNA gene analysis.

### 2.8. Rapid Miniaturized Biochemical Code for Preliminary Confirmation

For validation of the isolation protocol and rapid preliminary confirmation of* Lplant*-B80-like colonies during this preclinical trial, bacterial biochemical profiles were performed on random* Lplant*-B80-like isolates and other recovered LAB using the 4-hour incubation miniaturized BBL Crystal Anaerobic ID System (Becton Dickinson; 245010), which has 29 possible enzymatic test well reactions and reported overall reproducibility of 99.1% (96.2–100%) [16]. The resulting pattern of 29 reactions is converted into a ten-digit profile number that is used as the basis for identification stored in a BBL database, using a comparative approach and percentage of similarity/probability with respect to best hits. This system is designed to identify over 110 species of clinically relevant anaerobes, including the lactobacilli* L. acidophilus, L. casei, L. catenaforme, L. fermentum, L. jensenii, L. johnsonii, *and* L. rhamnosus* [16]. In this study isolates with non-*Lplant*-B80-like morphology or gram stain, or yielding unexpected BBL profiles, were further tested (15% of all isolates) using API 50CH miniaturize biochemical testing (bioMerieux) to further qualify the phenotypic profile probability and 16S rRNA sequencing for species level confirmation as the preferred molecular method [17, 18].

### 2.9. 16s rRNA Phylogenetic Analysis

Partial sequencing of 16S rRNA gene was conducted on selected isolates using primers (BSF8/20-F, AGA GTT TGA TCC TGG CTC AG; BSR534/18-R, ATT ACC GCG GCT GCT GGC) to amplify variable regions V1–V3 (500 bp) [19] following principles for LAB [20]. Assembled sequences were compared to the sequence of the orally administered isolate* Lplant*-B80, previously characterized [9]. 16s rRNA gene sequences were Blastn in NCBI datasets. A neighbour-joining method with a bootstrap consensus tree inferred from 500 replicates was created using MEGA5 [21]. Evolutionary distances were computed using the* p*-distance method and using 1st, 2nd, and 3rd codon and noncoding position, with ambiguous positions removed for each sequence pair [21].

### 2.10. Statistical Analysis

One-way ANOVA of the areas under the curves with multiple *t*-test comparisons for significant *F*-values was used to analyze the effect of treatments on fecal water content and the CFU number. Two-way ANOVA for repeated measures was used to identify treatment and day effect along with appropriate multiple means comparisons if *F*-value was significant. The effect of treatments on the recovery of* Lplant*-B80 was analyzed comparing the overall means of the log_10_ CFU and the average days of positive cultures per group. SAS Software was used (SAS Institute Inc, Cary, NC) (SAS, 1996). Nonparametric methods were used when assumptions were not fulfilled as described [22, 23]. Chi-square was used to compare proportions.

## 3. Results

### 3.1. Viability of* Lplant*-B80 during Storage on Freeze-Dried Pellets

Bacterial counts on frozen* Lplant*-B80 pellets confirmed the intended doses at administration, and culture of pellets stored at room temperature for several months showed that the CFU declined at a rate of about 1-2 log_10_ units after 6–12 months of storage. After 48 months, a high-dose pellet tested had 10 CFU per 100 mg of pellet. This simultaneous and prospective analysis shows that* L. plantarum* survive preparation and storage.

### 3.2. Oral Administration Yielded No Clinical Signs of Disease

**Figure 1:**
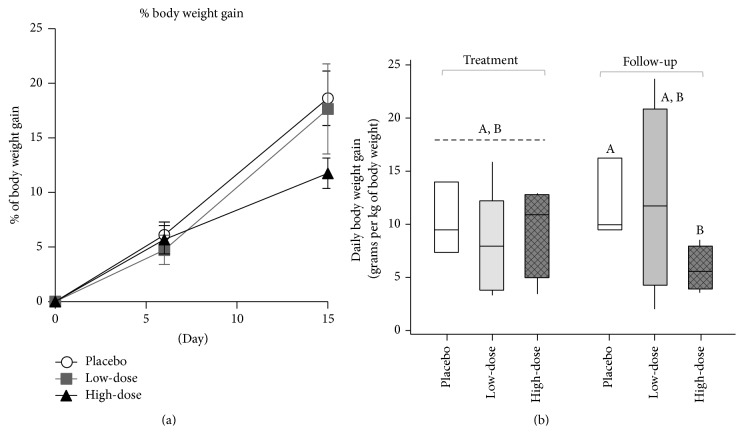
Body weight gain in neonatal calves during and after oral supplementation with* L. plantarum *strain B80. (a) Cumulative percentage of body weight gain. (b) Daily body weight gain efficiency. Data normalized to body weight at the beginning of each period. Distinct scripts (A, B) above the boxplots indicate pairwise difference (M-W, *P* = 0.051).

Oral administration of* L. plantarum* strain* Lplant*-B80 to calves resulted in no clinical signs of intestinal or systemic disease. Although watery feces (and diarrhea) are commonly observed in neonatal calves, no differences were observed for the cumulative numbers of days with loose stools during the study, or the fecal water content across the three treatment groups after the discontinuation of* Lplant*-B80 (6–15 d, ANOVA, *P* = 0.3). Body weight gain was similar across groups during the administration of* Lplant*-B80; however, it trended towards being lower in animals after receiving the high dose (follow-up period, 6–15 days, M-W, *P* = 0.051; Figure 1).

### 3.3. Rapid Biochemical Profile for Preliminary Confirmation of* Lplant*-B80

**Figure 2:**
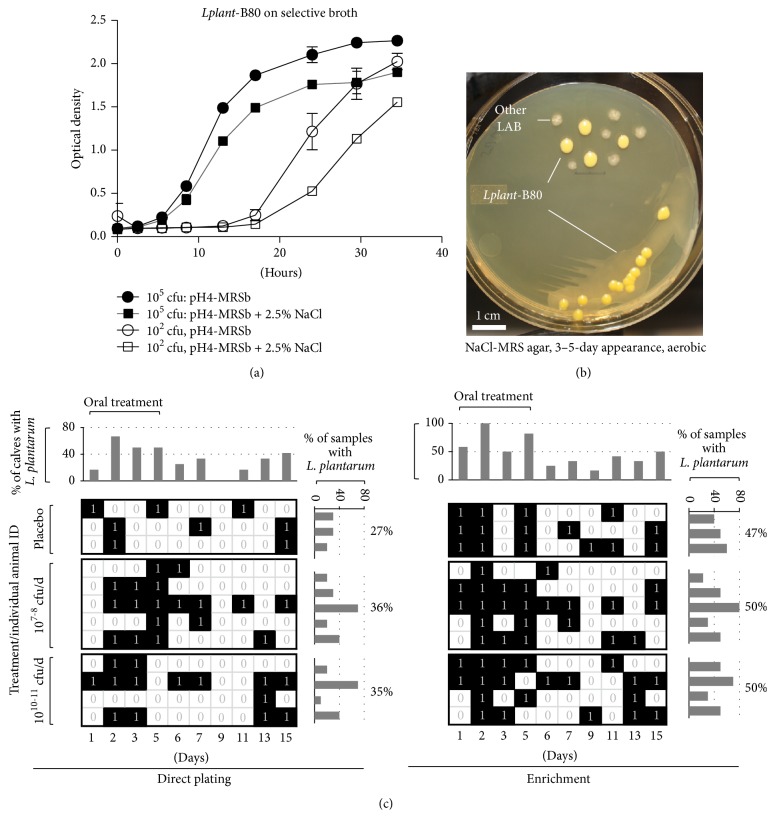
Selective microbial enumeration and colonization of* L. plantarum Lplant*-B80 following oral administration of low- and high doses in neonatal calves. (a) The use of optical density for quantification of bacterial growth in broth allowed the identification of optimal acid and salt concentrations to favor the optimal growth of as few as 10^2^ CFU of* Lplant*-B80 in an acid/salt-MRS broth, under aerobic conditions, preventing the growth of most previously tested LAB in our laboratory; see [9]. (b) Large distinct colonies for* Lplant*-B80 on MRS-salt agar, from a fraction of freeze-dried pellet stored for 48 months at room temperature and rehydrated with PBS. (c) Binary analysis of fecal colonization with* L. plantarum Lplant*-B80 before, during, and after administration. 1, recovered; 0, not recovered. Note similar overall recovery rate (histograms) across groups and high recovery during treatment period.

After testing 114 pure single* Lplant*-B80-like bacterial isolates, from feces and freeze-dried* Lplant*-B80 pellets, two unique rapid (4 hour) biochemical BBL profiles were identified and used for preliminary biochemical confirmation of the fed* Lplant*-B80 strain. Referent 24 h colonies of* L. plantarum* isolated from pellets fed to the calves (incubated in both plain and 2.5% NaCl-MRS agar, Figures 2(a) and 2(b)) yielded the BBL-ID code 011066-3-062 characterized by positive reactions for L-methionine, L-phenylalanine, L-leucine, L-alanine, L-isoleucine, p-n-p-*β*-D-galactoside, p-n-p-*β*-D-glucoside, p-n-p-*α*-D-glucoside, p-n-p-N-acetyl-glucosaminide, 4MU-*β*-D-cellobiopyranoside, furanose, and pyranose. Compared to* L. acidophilus* reference strain ATCC-314, our freshly grown* L. plantarum* isolates were negative on L-lysine, L-arginine, L-histidine, L-serine, and p-n-p-*α*-D-galactoside. Because identification of* Lplant*-B80 was facilitated by inspecting colonies after 3–5 days of incubation on the 2.5% NaCl-MRS agar, BBL testing of such aged colonies was also conducted. Of interest, the yielded BBL profile was slightly different (011046-3-000); therefore, testing of candidate colonies was always conducted with fresh 24 h subcultures of* Lplant*-B80 suspect isolates. Further testing of 15% of* Lplant*-B80 suspect isolates with API CH and 16s RNA analysis confirmed the recovery of* Lplant*-B80 isolates throughout the length of study and aided in identifying the distinctive colony morphology for* Lplant*-B80 isolates in the culture agar (Figures 2(a) and 2(b)).

### 3.4. Recovery of* Lplant*-B80 from Feces and Intestinal Mucosa

**Figure 3:**
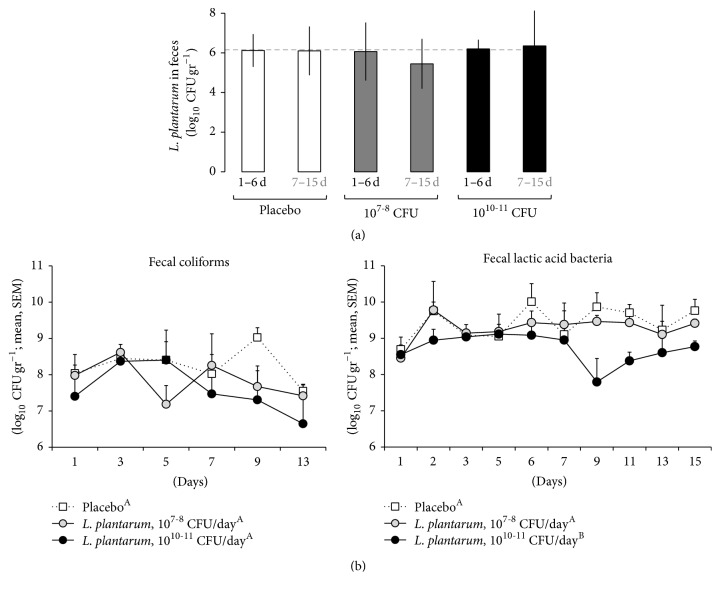
Quantitative effect of* L. plantarum* (*Lplant*-B80) administration on other fecal cultivable microbiota. (a) Selective enumeration of* L. plantarum* B80 based on phenotype and BBL biochemical profile. Data for the first 6 days of treatment and the follow-up period (7–15 days). Average ± SD. (b) Total fecal coliforms and lactic acid bacteria. High doses tended to lower fecal coliforms, but it significantly reduced lactic acid bacteria (areas under the curves, *P* < 0.01). Distinct capitalized superscripts (A, B) denote statistical differences.

This study involved the microbiological analysis of 120 fecal samples and 72 intestinal mucosal/lymphatic tissues, using direct and enrichment culture methods here developed, to enable the recovery of* Lplant*-B80-like isolates and test the effect of its administration on coliform and lactobacilli counts (*n* = 384 samples tested in >1,152 agar plates).* Using direct selective plating* and the BBL/morphological criteria*, L. plantarum* resembling* Lplant*-B80 was recovered from two of the 12 newborn calves prior to the administration of* Lplant*-B80 in animals at <24 h of age and from 27% (30 samples) from fecal samples in the placebo group at older ages (up to 15 days old).* Lplant*-B80-like bacteria were recovered in 35% of samples from* Lplant*-B80 treated animals*. Using enrichment broth* enabled a 2-fold increased recovery rate of* Lplant*-B80 in all groups (up to 47–50%; Figure 2(c)).* Lplant*-B80-like was most common in treated animals during the administration period; however, the amount of CFU of* L. plantarum* resembling* Lplant*-B80 in positive samples was unexpectedly similar across groups and over time (6 ± 1 log_10_/g, ANOVA, *P* > 0.5; Figure 3(a)). In adjusted regression analysis, there was a slight nonsignificant inhibitory effect on coliforms in the high-dose group (Figure 3(b)). For total lactic acid bacteria, high oral doses of* Lplant*-B80 significantly reduced fecal LAB until the end of the study (compared to placebo and low-dose, ANOVA, areas under the curve, *P* = 0.006 and 0.01; Figure 3(b)).

**Figure 4:**
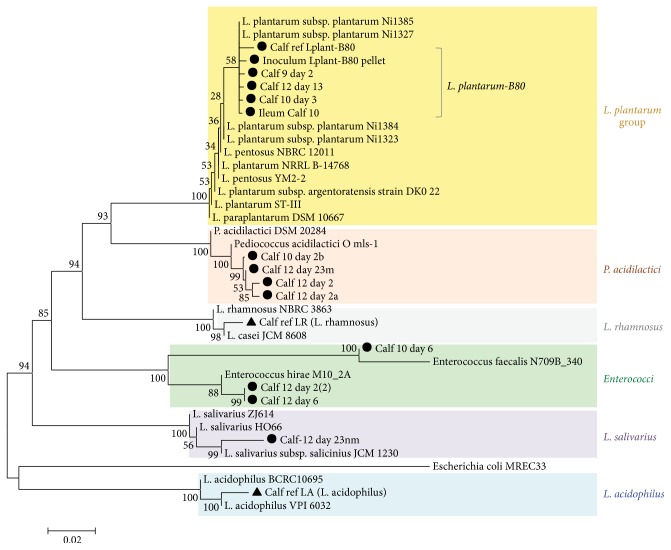
Neighbour-joining of 16S rRNA gene sequences of LAB from calves in this study. Numbers indicate percentage of replicate trees in which the associated taxa clustered together in the bootstrap test. Branch lengths are evolutionary distances, that is, units of the number of base differences per site. A total of 1614 positions were the final dataset. LAB isolates from this study (●) and internal laboratory references (▲). Remarkably the closest isolates in the NCBI collection are a group of isolates isolated from grass silage in Japan.

*Lactobacillus plantarum Lplant*-B80 (based on colony morphology and 100% similarity on BBL profile) was confirmed using single-colony PCR and Sanger sequencing of the 16s rRNA gene. Phylogenomic analysis of 16s rRNA gene data for various isolates in Figure 4 illustrates that* Lplant*-B80 was recovered from the intestinal mucosa in three animals: two from the ileum of low- and high-dose calves and one from the cecum of a high-dose calf, suggesting that this* L. plantarum* (*Lplant*-B80-like) strain is likely endemic at the farm and well adapted to the intestinal tract of neonatal calves. No other tissues yielded* Lplant*-B80. Overall, the strain used was uncommonly found in association with the mucosal surface (4.2%, 3 of 72 intestinal and lymph tissues) compared to its common presence in feces (50%, Chi-square, *P* < 0.001). Other pH-resistant LAB that grew aerobically on 2.5%-NaCl-MRS agar from feces with the selective protocol included* Pediococcus acidilactici*,* Enterococcus faecalis*,* Enterococcus hiriae*, and* L. salivarius*. The other LAB identified from intestinal mucosa in the selective medium were* L. pentosus*,* L. acidophilus*, and* L. salivarius* (Figure 4); however, none of them have the large yellowish discoloured colonies deemed characteristic of* Lplant*-B80 in the agar used in the present study (Figure 2(b)).

## 4. Discussion

Despite decades of observation that probiotics need to be given in high doses to exert positive health effects [24], little is known regarding the dose response effect of host-indigenous lactobacilli in intestinal health. Here we assessed the safety and dose-dependent colonization properties and inhibitory effect of calf-derived* L. plantarum* strain B80 in immune-competent colostrum-fed neonatal calves, using culture as the gold standard test for the colonization of this cultivable organism in this time series repeated experimental analysis. In this context, after designing a selective protocol based on NaCl (limit of detection, 10^3^* Lplant*-B80 in inoculated feces) and rapid miniaturized biochemistry, we discovered (i) that* L. plantarum* has a high natural occurrence in neonatal newborn calves (50% prevalence), (ii) that this* Lplant*-B80 strain has a presumptive natural persistence in the farm, since naturally occurring isolates resembled original strains isolated a year earlier from the ilea of calves [9], (iii) that at high doses administration is experimentally safe in calves with proper passive transfer of maternal immunity, and (iv) that the high doses decreased the abundance of total cultivable lactobacilli from the feces of calves, without decreasing total commensal coliform counts, despite reported inhibitory effects on pathogenic* E. coli* F5 and O157:H7 [9].

The lack of evidence for intestinal translocation to regional mesenteric lymph nodes indicates that* L. plantarum* strain B80 is contained within the intestinal tract of healthy calves. These experimental observations combined with the excellent aerobic growth and halotolerant nature of this microorganism (able to thrive at 2.5% NaCl) are assets that support recently illustrated biotechnological advantages of Halobacteria to be used as potential feed supplements, especially since* L. plantarum* species are known to thrive in plants and plant-derived fermented diets [25].

*Lactobacillus* administration has reduced faecal* Enterobacteriaceae* and anaerobic cocci in calves [26, 27]; however, earlier reports were limited to often describing the effect of primarily* L. acidophilus* or lactobacilli mixtures with minimal information on other cultivable fecal LAB [28], or coliform inhibition only observable when using whole-milk diets [29]. Our study documents the inhibitory effect that high doses of an indigenous* Lactobacillus* species had on other intestinal LAB. In contrast to human-derived* L. rhamnosus* and animal-derived* L. pentosus* studied in other animal species [30, 31], where higher fecal counts (“peak of colonization”) are observed during the oral administration (supplementation) period, the magnitude of shedding of this* L. plantarum* strain remained constant throughout our study suggesting that there was no intestinal overgrowth of* Lplant*-B80 during the study.

Despite the observed stable shedding of* Lplant*-B80, high doses resulted in the significant reduction of other intestinal LAB. These results seem paradoxical for* L. plantarum* since others have reported other lactobacilli changes in total intestinal LAB counts in the feces of calves [28, 32]. Of great interest, more recently, the administration of* L. plantarum* strain WCFS1 (resistant to preculture with NaCl) to yoghurt during production also resulted in the inhibition of other lactobacilli, specifically* L. delbrueckii* subsp.* bulgaricus* [33], for which the authors could not attribute a specific cause. The reduction of other LAB with high doses of* Lplant*-B80 in our culture-based study is unlikely to be due to ecological exclusion by competition or displacement alone secondary to overgrowth of* Lplant*-B80 because its shedding was similar across treatment groups, although local exclusion is possible [34]. More likely is the inhibitory effect via the production of plantaricins (bacteriocins, i.e., antimicrobial peptide targeted against Gram-positive bacteria, including lactobacilli, and Gram-negative bacteria like* Salmonella* spp. and* E. coli*) [35, 36], but that remains a hypothesis that needs to be further tested in vivo. Increasingly there is evidence that immune-mediated modulation could occur in association with specific pathogens and cytokines or receptors via modulation of immune cells or the dose-dependent presence of cell wall components. Metabolic regulatory mechanisms with the gut microbiota are also possible [37, 38]. Modulation of LAB via community composition changes is under investigation using metagenomics.

*Lactobacillus plantarum* strains are increasingly promising as potential probiotics as aerotolerant strains that could be included as additives in pelletized or fermented feeding [33, 39]. The promising role of probiotics for modulation of animal intestinal health, control of zoonotic pathogens, and body gain efficiency requires deeper understanding of the effect of probiotics on other LAB. Although this study might indicate that the administration of this bacterium could result in a statistical reduction of the growth efficiency with high doses during a short period of time, it is important to interpret the findings with caution. Longer and larger clinical trials are required to validate this observation since compensatory overgrowth in neonates is possible, minimizing the relevance of this potential side effect in the long term.

Lactobacilli are generally regarded as beneficial, but under different circumstances (not due to the administration of microbial dietary additives), a net reduction of fecal lactobacilli has been reported in other animal species and humans as a consequence of stressing factors, which together are believed to carry an additive risk for negative health effects [40]. In monkeys, early maternal separation as stressor factor has resulted in increased intestinal infection risks with* Campylobacter jejuni* and* Shigella flexneri* and reduced counts of fecal lactobacilli [41, 42]. Of experimental and clinical interest, prenatal acoustic stress inflicted to monkeys during gestation resulted in newborn monkeys having reduced fecal lactobacilli for several weeks after birth [42]. Studies in mice have shown somewhat similar results depending on the stressor tested. Reduced fecal lactobacilli have been induced by stressing mice using constant shaking of their housing cage, by overnight physical restrain in confined spaces, by social stress or conflict, or by housing mice without bedding material. Water restriction, although deemed stressful, has not always reproduced such reduction in murine fecal lactobacilli [43–45]. Stress was also shown to reduce the number of tissue-associated lactobacilli in the colon of experimental mice [46] indicating that stress-mediated immunomodulation could be detrimental for the commensal interaction of the host with lactobacilli. In humans, studies with college students during the week of final exams have shown less lactobacilli in the stools compared to fecal concentrations the first week of the semester [47]. Although salivary cortisol indicated that stress was associated with lactobacilli reductions during exam times, it is worthwhile to notice that dietary anomalies also occurred during final exams, which could independently modulate the gut microbiota and possibly reduce the concentrations of fecal lactobacilli.

Stressor-induced immunomodulation is plausible as part of the gut-axis hypothesis to connect the host wellbeing to the gut microbiota and the reduction of lactobacilli and bifidobacteria in the aforementioned scenarios. However, in the present study, the daily clinical examination of the neonatal calves, and the assessment of appetite, animal behaviour, and attitude towards the milk feeding and the environment indicated that stress or depression are unlikely the cause of lactobacilli reduction in our report. The only difference in treatment between the closest groups (high versus low-dose* Lplant*-B80) was 1000-fold more* L. plantarum* CFUs, which is unlikely to have been perceived by the animals as a stressing factor. As the animal handlers were also blinded to the treatment codes, it is also unlikely that bias existed in the form of managerial/husbandry-induced stress only in the high-dose group of calves. Experimentally, mice stressed are more likely to have increased susceptibility to coliform intestinal infections (e.g.,* Citrobacter rodentium*) [48], which can be reverted by administering* Lactobacillus reuteri* [49]. In our study, the reduction of cultivable lactobacilli in the high-dose* Lplant*-B80 group did not result in an increased load of fecal coliforms or signs of intestinal disease in the neonatal calves, indicating that previously observed associations between stress, increased risk of infections, and reduced fecal lactobacilli do not exactly apply to our findings.

In a series of studies required for the validation of commercial probiotics, this report represents the first in vivo safety and colonization blinded-placebo study in calves for this strain. Aware of the technical difficulties and limitations of 16S microbiome in speciation of cultivable bacteria within the* Lactobacillus* genus, our results were deemed to be optimal in identifying and assessing the impact of feeding cultivable* Lplant*-B80-like bacteria to neonatal animals, as supported by single-colony 16S rDNA gene Sanger sequencing. This study provides preliminary statistical evidence that the significant reduction of fecal LAB in the calves in the high-dose group (assessed using valid repeated-measure statistical methods) is independent of the concentration of* L. plantarum*-B80 like bacteria in the feces.

In conclusion, our findings indicate that high doses of halotolerant gut-indigenous* L. plantarum-*B80 reduce cultivable lactobacilli in newborn calves without increasing its species abundance and without exerting overly signs of clinical disease or bacterial translocation to the regional mesenteric lymph nodes. Our findings do not necessarily support the assumption that reduced lactobacilli always correlate with coliform proliferation and risk of intestinal infections or disease. Mechanistic studies using conventional and germ-free hosts, aided with fluorescent chromosomal reporting probes, and clinical trials for specific end-point health outcomes are warranted to determine the impact of reduced LAB on health and the potential value of using high doses and halotolerance as criteria to select future commercially suitable probiotic strains.
